# Ectopic gut colonization: a metagenomic study of the oral and gut microbiome in Crohn’s disease

**DOI:** 10.1186/s13099-021-00409-5

**Published:** 2021-02-25

**Authors:** Shijia Hu, Eileen Png, Michelle Gowans, David E. H. Ong, Paola Florez de Sessions, Jie Song, Niranjan Nagarajan

**Affiliations:** 1 Discipline of Orthodontics and Paediatric Dentistry, Faculty of Dentistry, National University of Singapore, 9 Lower Kent Ridge Road, Singapore, 119085 Singapore; 2 Genome Institute of Singapore, Agency for Science, Technology and Research (A*STAR), 60 Biopolis St, Singapore, 138672 Singapore; 3 Division of Gastroenterology & Hepatology, National University Hospital, 5 Lower Kent Ridge Rd, Singapore, 119074 Singapore; 4 Yong Loo Lin School of Medicine, National University of Singapore, Singapore, Singapore

**Keywords:** Crohn’s disease, Gastrointestinal microbiome, Oral microbiome, Metagenomics

## Abstract

**Background:**

This study aims to characterize, the gut and oral microbiome in Asian subjects with Crohn’s disease (CD) using whole genome shotgun sequencing, thereby allowing for strain-level comparison.

**Methods:**

A case–control study with age, sex and ethnicity matched healthy controls was conducted. CD subjects were limited to well-controlled patients without oral manifestations. Fecal and saliva samples were collected for characterization of gut and oral microbiome respectively. Microbial DNA were extracted, libraries prepared and sequenced reads profiled. Taxonomic diversity, taxonomic association, strain typing and microbial gene pathway analyses were conducted.

**Results:**

The study recruited 25 subjects with CD and 25 healthy controls. The oral microbe *Streptococcus salivarius* was found to be enriched and of concordant strains in the gut and oral microbiome of Crohn’s disease subjects. This was more likely in CD subjects with higher Crohn’s Disease Activity Index (184.3 ± 2.9 vs 67.1 ± 82.5, p = 0.012) and active disease status (Diarrhoea/abdominal pain/blood-in-stool/fever and fatigue) (p = 0.016). Gut species found to be significantly depleted in CD compared to control (Relative abundance: Median[Range]) include: *Faecalibacterium prausnitzii* (0.03[0.00–4.56] vs 13.69[5.32–18.71], p = 0.010), *Roseburia inulinivorans* (0.00[0.00–0.03] vs 0.21[0.01–0.53], p = 0.010) and *Alistipes senegalensis* (0.00[0.00–0.00] vs 0.00[0.00–0.02], p = 0.029). While *Clostridium nexile* (0.00[0.00–0.12] vs 0.00[0.00–0.00], p = 0.038) and *Ruminococcus gnavus* (0.43[0.02–0.33] vs 0.00[0.00–0.13], p = 0.043) were found to be enriched. *C. nexile* enrichment was not found in CD subjects of European descent. Microbial arginine (Linear-discriminant-analysis: 3.162, p = 0.001) and isoprene (Linear-discriminant-analysis: 3.058, p < 0.001) pathways were found at a higher relative abundance level in gut microbiome of Crohn’s disease.

**Conclusions:**

There was evidence of ectopic gut colonization by oral bacteria, especially during the active phase of CD. Previously studied gut microbial differences were detected, in addition to novel associations which could have resulted from geographical/ethnic differences to subjects of European descent. Differences in microbial pathways provide possible targets for microbiome modification.

## Background

Inflammatory bowel diseases (IBD) are a group of digestive tract disease that affect millions of people worldwide. There are signs that incidence rates of IBD are increasing and presenting earlier in life [1]. IBD is divided into 2 main disease processes: Crohn’s Disease (CD), which may affect any segment of the gastro-intestinal tract, and Ulcerative Colitis (UC), which is limited to the large intestine [2]. Patients with CD frequently present with severe abdominal pain, fever, and clinical signs of bowel obstruction or diarrhea [3]. Inflammation modulation is the mainstay of medical management and ranges from the use of anti-inflammatories, to corticosteroids and immunomodulators [4]. In severe cases, surgery may be required. Unfortunately, the need for surgery has not decreased despite advancements in diagnostic and treatment protocols [5].

Despite theorizing that CD arises from an impaired interaction between commensal microbiome and the human host, the distinction between primary driver events and secondary occurrences remains murky. However, the recent focus on gut microbiome dysbiosis in CD has led to the discovery of new diagnostic and therapeutic directions [6]. Murine models have shown that the disease only manifests in susceptible genotypes and is driven by microbial dysbiosis [7], while human studies have found a general decrease in alpha diversity with clade-specific changes in CD patients such as increased *Enterobacteriaceae* and decreased Firmicutes [8, 9].

One of the limitations of previous microbiome studies has been the sequencing strategy. Most of the studies employed 16S rRNA sequencing, which limits their findings to bacteria at the genus level. This gap can be addressed with whole genome shotgun (WGS) sequencing, which is able to detect the presence of microbes with better accuracy and provide data at species level [10]. Additionally, most of these studies were in western populations and with subjects of European descent. This limited the scope of previous results as ethnicity, eating habits and living environment are variables that affect the gut microbiome [11].

Previous work on the oral-gut axis has demonstrated a connection between oral inflammation and its contribution to gut inflammation in animal models. Oral pathobiont-reactive inflammatory cells arising from oral inflammation were found to migrate to the gut, promoting and contributing to colitis [12]. The effect of the oral microbiome on CD is by comparison relatively understudied; however, its impact cannot be ignored [13]. A recent study found that oral *Klebsiella* can colonize the gut and result in severe gut inflammation in susceptible individuals, thereby exacerbating inflammatory disease [14]. Another study found increased presence of species found abundantly in oral communities in the gut microbiomes of CD subjects having diarrhea, suggesting that the oral cavity may serve as a reservoir for opportunistic gut pathogens [15].

Previous studies examining the oral microbiome were also obfuscated by having different sampling sites, such as sampling from the tongue [16], plaque [17] and saliva [18, 19]. Of these sampling sites, the salivary microbiome appears to offer the most diagnostic value without the excessive influence of local factors [20]. These studies found enrichment of *Veillonellaceae* and depletion of *Haemophilus* in CD subjects, highlighting the diagnostic value of the oral microbiome [18, 19]. Moreover, as a diagnostic tool, saliva samples are non-invasive unlike colonoscopy, and easy to collect at any time unlike fecal samples.

This study aims to characterize, for the first time, the oral and gut microbiome in Asian subjects with CD using whole genome shotgun technique. A case–control analysis was conducted with matched healthy controls to investigate the presence of altered community structure and different community ecotypes in the oral and gut microbiomes of a mixed Asian population consisting of Han Chinese, Malay and Indian.

## Results

The study recruited 25 subjects with CD and 25 healthy controls who were age, sex and ethnicity matched. The subjects’ demographics, clinical and oral conditions are summarized in Table 1. Most of the subjects were well controlled with Crohn’s Disease Activity Index (CDAI) scores lower than 150. There were no differences found between the caries and periodontal status between the control and CD groups. To examine the oral and gut microbiome, 50 saliva and 50 fecal samples were processed, DNA extracted and sequenced using whole genome shotgun sequencing.

**Table 1:** Demographics, clinical and oral conditions of subjects (n = 50)

	Crohn's disease (CD)	Healthy controls (HC)
Mean age^*^ (Range)	40 (23–67)	40 (21–66)
Race		
Han Chinese	13	13
Malay	3	3
Indian	9	9
Sex		
Male	10	10
Female	15	15
Oral condition		
Mean DMFT^^^	6.5 ± 7.0	4.6 ± 5.0
Periodontitis present	5	5
Smoking		
Non-smoker	20	21
Previous/current smoker	5	4
CD condition		
Length of diagnosis in years (range)	7.8 (1–20)	
Crohn’s Disease Activity Index (CDAI)	86.2 ± 87.1	
Current disease activity: Active (Diarrhoea, Abdominal pain, Blood-in-stool, fever and fatigue)	10	
Drug regimen^#^		
Steroids/Anti-inflammatory (Mesalazine)	8	
Azathioprine	13	
Biologics	11	
Others (Tacrolimus, sulphasalazine)	2	

^*^Matched controls are ± 3 years

^^^p-value = 0.29

^#^some subjects are on multiple drug regimen

### Gut microbiome profile and differentially abundant species

**Fig. 1:**
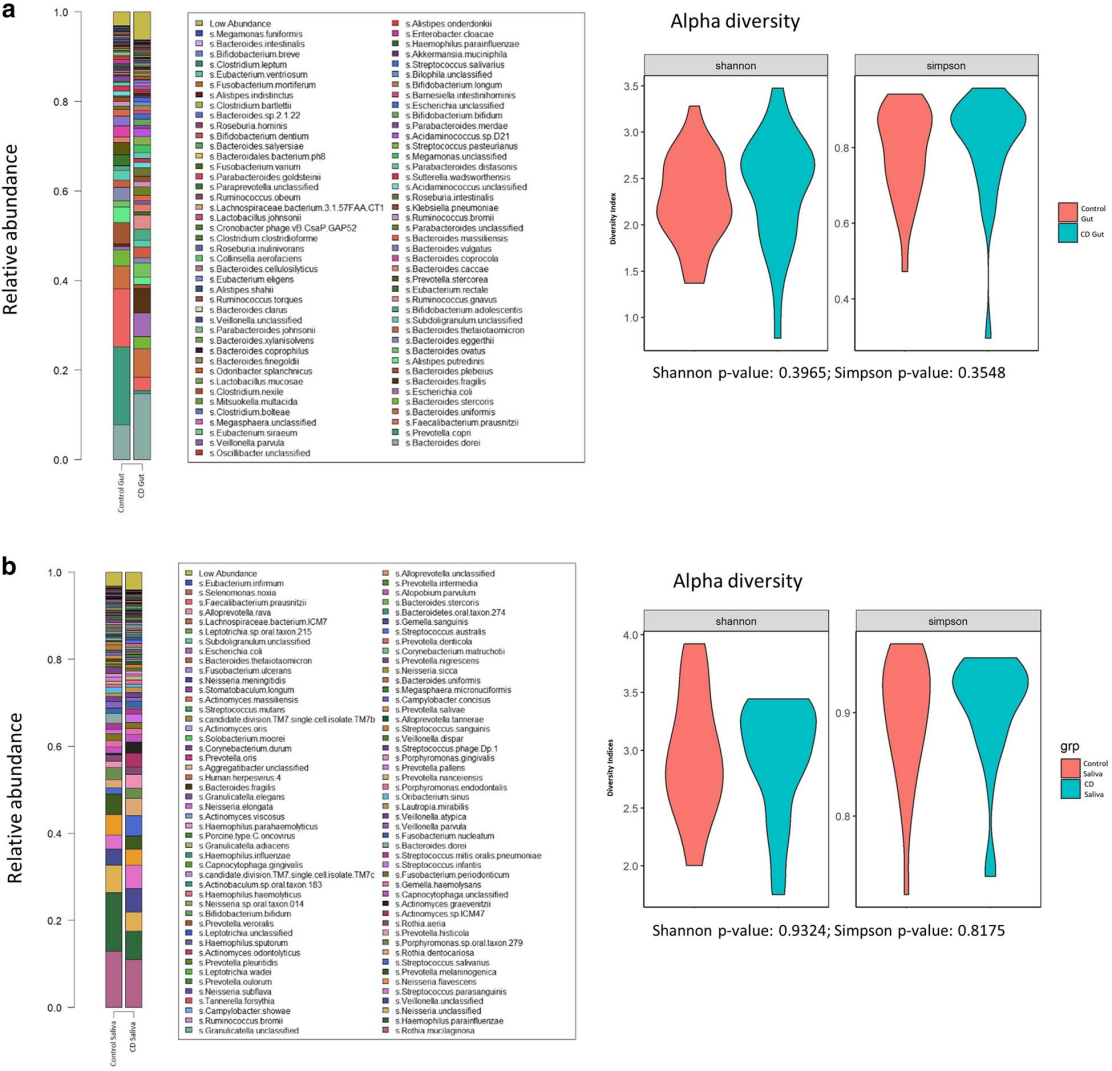
Microbiome diversity at species level in Healthy (Control) vs Crohn’s Disease (CD). **a** Gut microbiome. **b** Salivary Microbiome

Many commensal gut microbial species were found present at high abundances (> 5%) in both CD and healthy controls. These include *Bacteroides dorei* (11.2%), *Prevotella copri* (9.1%), *Faecalibacterium prausnitzii* (8.0%), and *Bacteroides uniformis* (5.8%) (Fig. 1a). There was no difference noted in alpha diversity at species level.

**Fig. 2:**
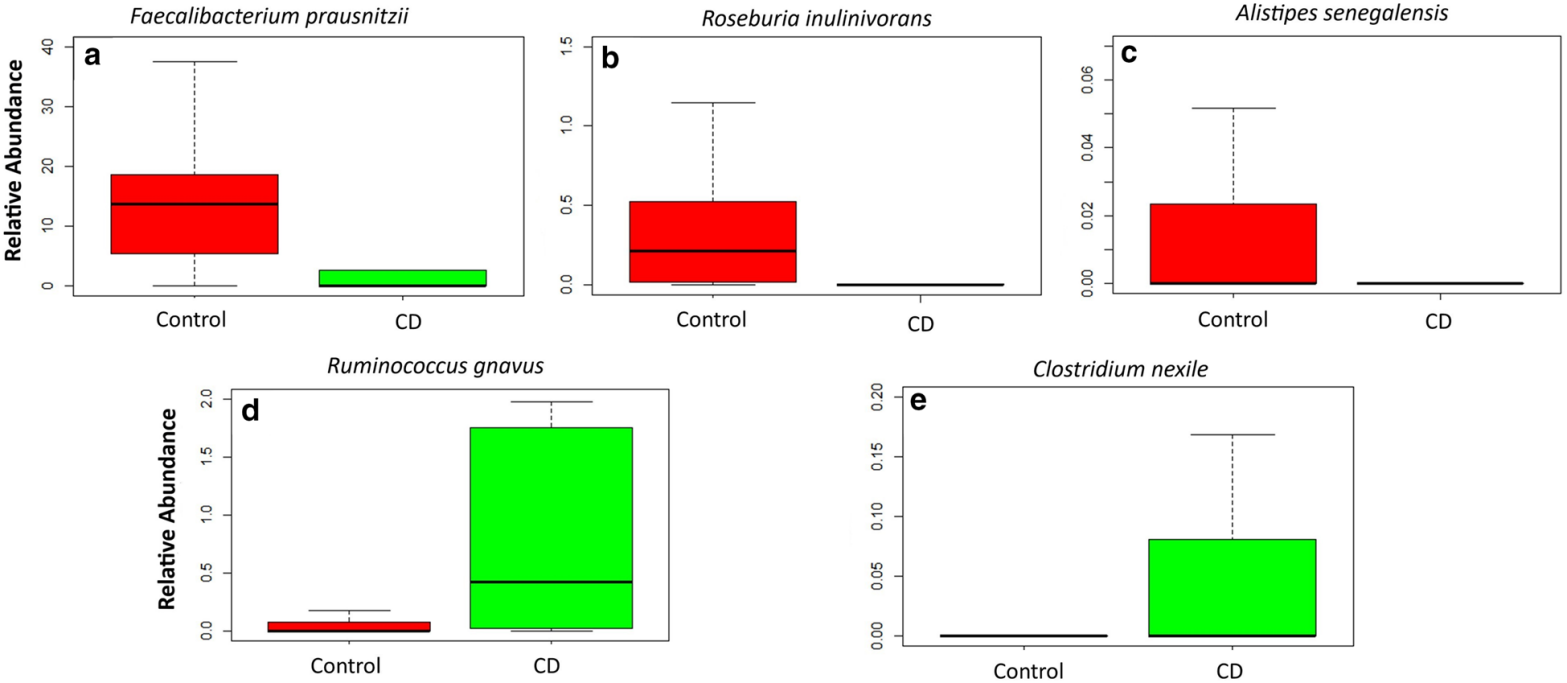
Gut microbiome species relative abundance in Healthy (Control) vs Crohn’s Disease (CD). **a** *Faecalibacterium prausnitzii*. **b** *Roseburia inulinivorans*. **c** *Alistipes senegalensis*. **d** *Ruminococcus gnavus*. **e** *Clostridium nexile*

The species that were found to be significantly depleted (relative abundance) in CD compared to control include: *F. prausnitzii* (Median [Range]: 0.03 [0.00–4.56] vs 13.69 [5.32–18.71], p = 0.010), *Roseburia inulinivorans* (Median [Range]: 0.00 [0.00–0.03] vs 0.21 [0.01–0.53], p = 0.010) and *Alistipes senegalensis* (Median [Range]: 0.00 [0.00–0.00] vs 0.00 [0.00–0.02], p = 0.029), while *Clostridium nexile* (Median [Range]: 0.00 [0.00–0.12] vs 0.00 [0.00–0.00], p = 0.038) and *Ruminococcus gnavus* (Median [Range]: 0.43 [0.02–0.33] vs 0.00 [0.00–0.13], p = 0.043) were found to be significantly enriched in subjects with CD compared to control (Fig. 2) (Table 2).

**Table 2:** Species level differences between Crohn's disease and Control

Depleted in crohn's disease			Enriched in crohn's disease		
Species	p-value	Adjusted p-value	Species	p-value	Adjusted p-value
Gut					
Faecalibacterium prausnitzii*	< 0.001	0.010	Clostridium nexile*	< 0.001	0.038
Roseburia inulinivorans*	< 0.001	0.010	Ruminococcus gnavus*	0.001	0.043
Alistipes senegalensis*	< 0.001	0.029	*Bifidobacterium breve*	0.001	0.060
* Bacteroides faecis*	0.001	0.060	*Escherichia unclassified*	0.003	0.105
* Coprococcus catus*	0.002	0.083	*Escherichia coli*	0.004	0.115
* Ruminococcus callidus*	0.002	0.099	*Anaerostipes unclassified*	0.005	0.115
* Eubacterium rectale*	0.003	0.100	*Streptococcus anginosus*	0.005	0.115
* Parabacteroides merdae*	0.005	0.115	*Streptococcus pasteurianus*	0.005	0.115
* Bacteroides vulgatus*	0.006	0.125	*Coprobacillus unclassified*	0.009	0.149
* Eubacterium eligens*	0.007	0.140	*Atopobium parvulum*	0.010	0.149
* Dorea longicatena*	0.007	0.140	*Fusobacterium nucleatum*	0.010	0.149
* Megamonas hypermegale*	0.010	0.149	*Blautia producta*	0.021	0.208
* Alistipes putredinis*	0.010	0.149	*Erysipelotrichaceae bacterium*	0.021	0.208
* Catenibacterium mitsuokai*	0.010	0.149	*Morganella morganii*	0.021	0.208
* Bacteroides coprocola*	0.012	0.163	*Streptococcus macedonicus*	0.021	0.208
* Paraprevotella unclassified*	0.013	0.163	*Proteus mirabilis*	0.021	0.208
* Coprococcus comes*	0.013	0.163	*Veillonella atypica*	0.026	0.232
* Peptostreptococcaceae unclassified*	0.014	0.170	*Acidaminococcus fermentans*	0.041	0.268
* Bacteroides plebeius*	0.018	0.208	*Acidaminococcus sp D21*	0.041	0.268
* Eubacterium ventriosum*	0.021	0.208	*Clostridium innocuum*	0.041	0.268
* Bacteroides stercoris*	0.023	0.219	*Lachnospiraceae bacterium*	0.041	0.268
* Alistipes finegoldii*	0.024	0.222	*Lactobacillus gasseri*	0.041	0.268
* Alistipes shahii*	0.025	0.228	*Lactobacillus mucosae*	0.041	0.268
* Bacteroides nordii*	0.027	0.236	*Peptostreptococcus anaerobius*	0.041	0.268
* Odoribacter splanchnicus*	0.028	0.236	*Prevotella histicola*	0.041	0.268
* Eubacterium siraeum*	0.031	0.260	*Scardovia wiggsiae*	0.041	0.268
* Holdemania unclassified*	0.037	0.268	*Streptococcus mutans*	0.041	0.268
* Lachnospiraceae bacterium 5_1_63FAA*	0.038	0.268	*Rothia dentocariosa*	0.043	0.273
* Barnesiella intestinihominis*	0.049	0.297	*Clostridium ramosum*	0.048	0.297
* Roseburia hominis*	0.049	0.297			
Saliva					
* Bacteroides uniformis*	< 0.001	0.117	*Human herpesvirus 4*	0.002	0.117
* Bacteroides dorei*	0.001	0.117	*Streptococcus anginosus*	0.002	0.117
* Haemophilus parainfluenzae*	0.003	0.128	*Streptococcus oligofermentans*	0.002	0.117
* Subdoligranulum unclassified*	0.023	0.526	*Streptococcus vestibularis*	0.002	0.117
* Prevotella salivae*	0.033	0.526	*Streptococcus cristatus*	0.004	0.134
* Prevotella intermedia*	0.039	0.526	*Peptostreptococcus stomatis*	0.010	0.326
			*Streptococcus salivarius*	0.020	0.526
			*Actinomyces sp. ICM47*	0.021	0.526
			*Actinomyces naeslundii*	0.032	0.526
			*Stomatobaculum longum*	0.033	0.526
			*Actinomyces georgiae*	0.041	0.526
			*Lactobacillus fermentum*	0.041	0.526
			*Olsenella_uli*	0.041	0.526
			*Oribacterium sp. Oral taxon 078*	0.041	0.526
			*Parvimonas micra*	0.041	0.526
			*Slackia unclassified*	0.041	0.526
			*Streptococcus peroris*	0.046	0.564

*Adjusted p-value < 0.05

**Fig. 3:**
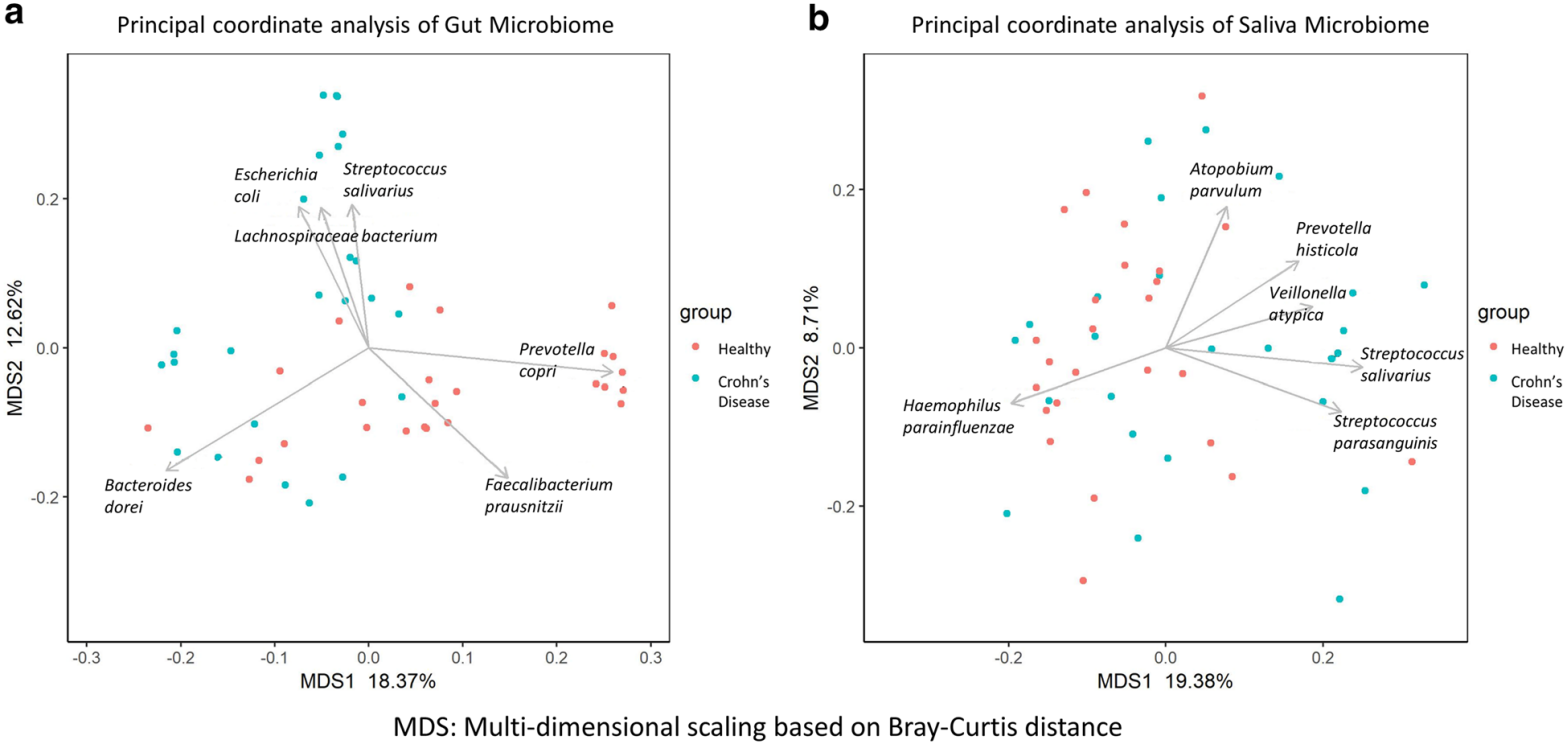
Principal coordinate analysis of microbiome in Healthy (Control) vs Crohn’s Disease (CD). **a** Gut microbiome. **b** Salivary Microbiome. The arrows drawn are for the top 6 most significant species at p-value ≤ 0.0025

Principal coordinate analysis (Fig. 3a) revealed visually-derived groups of healthy controls driven by *Prevotella copri* and *F. prausnitzii*, while a group of CD subjects was characterized by *Escherichia coli*, *Streptococcus salivarius* and *Lachnospiraceae* (Wilcoxon test, p-value ≤ 0.0025)*.*

### Oral microbiome profile and differentially abundant species

The oral microbiome was less dominated by specific species as compared to the gut microbiome. Microbes present at high abundance include *Rothia mucilaginosa* (12.0%), *Haemophilus parainfluenzae* (10.3%), and *Neisseria sp.* (5.7%). There was also no difference noted in alpha diversity at species level between CD and control (Fig. 1b).

Interestingly, several *Streptoccocus sp.* and *Human Herpesvirus 4* were found to be enriched and *Bacteroides sp* found to be depleted in subjects with CD; however, they were not significantly different after multiple testing correction (Table 2).

Principal coordinate analysis conducted on the species found in the oral microbiome did not reveal distinct visual clusters based on CD status. The microbiome also did not cluster based on oral conditions such as caries or periodontal disease (Fig. 3b).

### Relationship between gut and oral microbiome

**Fig. 4:**
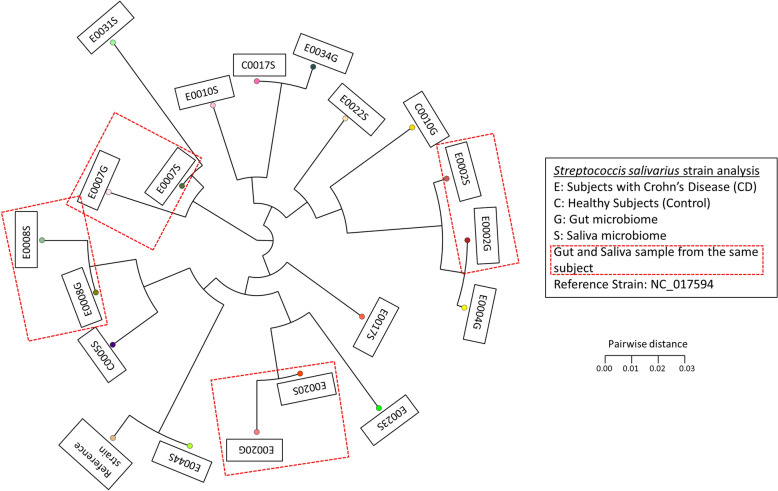
Phylogenetic tree plot of *Streptococcus salivarius* strain analysis for all Gut and Saliva samples

Out of the 50 pairs of gut and oral samples, *Streptoccous salivarius* were detected in 19 libraries; 7 gut and 12 oral samples. Among these, *S. salivarius* was detected in both the gut and oral samples in 4 subjects, all with CD. A strain typing analysis based on multiple sequence alignment of marker genes of *S. salivarius* in the subjects showed that the gut and saliva strains from the same subject clustered in close distance despite the samples being sampled separately (Fig. 4 and Additional file 1: Table S1). This suggests the *S. salivarius* found in the saliva and gut were colonised by similar strains. This finding was not seen in the healthy controls.

Clinical characteristics of the subjects with concordant gut and oral S. salivarius strains were compared (Table 3). Subjects with higher CDAI scores (< 150) indicating active disease were significantly more likely to have concordant strains of *S. salivarius* (*p* = 0.012). This was also reflected in subjects with signs and symptoms of active disease (Diarrhoea/abdominal pain/blood-in-stool/fever and fatigue) at the time of sampling (*p* = 0.016). Other clinical characteristics such as age, sex, race and oral conditions did not result in having more likely concordant gut and oral strains. Additionally, the use of proton pump inhibitors did not result in having more likely concordant gut and oral strains.

**Table 3:** Clinical characteristics of subjects with concordant gut and oral *S. salivarius* strains

	CD subjects with concordant strains (n = 4)	CD subjects with no concordant strain (n = 21)	*p*-value
Crohn’s Disease Activity Index (CDAI)^#^	184.3 ± 2.9	67.1 ± 82.5	*0.012
Disease status			
Active disease at time of sampling (Diarrhoea/abdominal pain/blood-in-stool/fever and fatigue)	4	6	*0.016
Inactive disease at time of sampling	0	15	
Proton Pump Inhibitor use			1.000
Yes	1	7	
No	3	14	
Mean age^#^	38 ± 14	40 ± 14	0.774
Race			0.056
Han Chinese	4	9	
Malay	0	3	
Indian	0	9	
Sex			0.142
Male	3	7	
Female	1	14	
Oral condition			0.661
DMFT^#^	5.0 ± 4.2	6.8 ± 7.4	
Periodontitis			0.451
Present	1	4	
Absent	3	17	

All Fisher-Exact test except ^#^T-test

*p-value < 0.05

### Differentially abundant microbial pathways

The HUMAnN2 analysis detected 395 pathways in the gut microbiome, of which 33 had Linear-discriminant-analysis (LDA) scores ≥ 3 (Table 4). The top 4 pathways with higher relative abundance levels found in CD compared to control were related to the biosynthesis of arginine: L-arginine biosynthesis II (acetylcycle) (LDA = 3.162, p = 0.001), L-ornithine biosynthesis (LDA = 3.084, p = 0.001), L-arginine biosynthesis I (via L-ornithine) (LDA = 3.077, p = 0.006) and L-arginine biosynthesis IV (archaebacterial) (LDA = 3.059, p = 0.008). The other higher relative abundance level pathways involve isoprene biosynthesis (2 pathways) (LDA = 3.058 and 3.042, p =  < 0.001 and < 0.001) and glycolysis (2 pathways) (LDA = 3.038 and 3.023, p =  < 0.001 and 0.001). While the lower relative abundance level pathways found in CD compared to control include the S-adenosyl-L-methionine cycle I (LDA = 3.550, p < 0.001), UMP biosynthesis (LDA = 3.434, p < 0.001) and 2 L-lysine biosynthesis pathways (LDA = 3.471 and 3.421, p =  < 0.001 and 0.001).

**Table 4:** Microbial pathways with LDA effect > 3

Most common in control group			Most common in crohn's disease group		
Pathway	LDA effect	P-value	Pathway	LDA effect	P-value
Gut					
PWY 6151 S-adenosyl-L-methioninecycle I	3.550	< 0.001	ARGSYNBSUB PWY L-arginine biosynthesis II (acetylcycle)	3.162	0.001
PWY 5097 L-lysine biosynthesis VI	3.471	< 0.001	GLUTORN PWY L-ornithine biosynthesis	3.084	0.001
PWY 5686 UMP biosynthesis	3.434	< 0.001	ARGSYN PWY L-arginine biosynthesis I (via L-ornithine)	3.077	0.006
PWY 2942 L-lysine biosynthesis III	3.421	0.001	PWY-7400 L-arginine biosynthesis IV (archaebacterial)	3.059	0.008
PWY 6700 queuosine biosynthesis	3.414	0.002	PWY 6270 isoprene biosynthesis I	3.058	< 0.001
NONMEVIPP PWY methylerythritol phosphate pathway I	3.414	< 0.001	PWY 7560 methylerythritol phosphate pathway II	3.042	< 0.001
PWY 6737 starch degradation V	3.335	0.003	GLYCOLYSIS glycolysis I from glucose-6-phosphate	3.038	< 0.001
PWY 7219 adenosine ribonucleotides de novo biosynthesis	3.296	0.001	PWY66 400 glycolysis VI (metazoan)	3.023	0.001
PWY 6386 UDP N-acetylmuramoyl pentapeptide biosynthesis II (lysine containing)	3.234	0.003	PWY 5484 glycolysis II from fructose-6-phosphate	3.017	0.000
PWY 7221 guanosine ribonucleotides de novo biosynthesis	3.219	0.004			
PEPTIDOGLYCANSYN PWY peptidoglycan biosynthesis I (meso-diaminopimelate containing)	3.212	0.003			
ILEUSYN PWY L-isoleucine biosynthesis I (from threonine)	3.212	0.006			
VALSYN PWY L-valine biosynthesis	3.212	0.006			
PWY 6387 UDP N-acetylmuramoyl pentapeptide biosynthesis I (meso-diaminopimelate containing)	3.170	0.003			
BRANCHED CHAIN AA SYN PWY superpathway of branched aminoacid biosynthesis	3.132	0.008			
GALACTUROCAT PWY D-galacturonate degradation I	3.113	< 0.001			
PWY 6897 thiamin salvage II	3.104	0.001			
PWY 7111 pyruvate fermentation to isobutanol engineered	3.098	0.015			
PWY 5973 cis-vaccenate biosynthesis	3.096	< 0.001			
GALACT GLUCUROCAT PWY superpathway of hexuronide and hexuronate degradation	3.087	0.001			
GLUCUROCAT PWY superpathway of beta D-glucuronide and D-glucuronate degradation	3.078	0.001			
PWY 5103 L-isoleucine biosynthesis III	3.062	0.012			
PWY 6507 4-deoxy-L-threo-hex-4-enopyranuronate degradation	3.040	0.001			
PWY 7242 D-fructuronate degradation	3.019	0.003			
Saliva					
FASYN INITIAL PWY superpathway of fatty acid biosynthesis initiation (E-coli)	3.036	0.002	PWY66 409 superpathway of purine nucleotide salvage	3.035	0.018

In the oral microbiomes, 365 pathways were detected, of which 2 had LDA scores ≥ 3 (Table 3). In CD, the superpathway of purine nucleotide salvage was found to have a higher relative abundance level while the superpathway of fatty acid biosynthesis initiation (*E. coli*) had a lower relative abundance level than the control group.

## Discussion

This study represents a metagenomic insight into the oral and gut microbiome in CD patients, conducted in an Asian population, consisting subjects of Chinese, Indian and Malay descent (Table 1), which may have innate differences in gut and oral microbiome. Although some studies have examined the gut microbiome in CD [10, 21], none of the previous studies on the oral microbiome employed the shotgun metagenomic technique used in this study. Furthermore, this is the first metagenomic study of matched oral and gut samples in CD subjects, allowing matching of the gut and oral microbiome at the strain level.

Crucially, this study found a cluster of CD subjects whose gut microbiome were characterized by *S. salivarius*, a prominent oral microbe. In health, *Streptococcus* genus typically constitute less than 4% of gut microbiome [13], thus it was note-worthy to find *S. salivarius* enriched in the gut microbiome of CD subjects. Although the ectopic colonization of oral bacteria such as *Klebsiella pneumoniae* has been found to induce intestinal inflammation and can result in the progression of CD [14], it was only demonstrated in mouse models. Other studies suggesting roles in CD by oral bacteria were largely based on finding typically oral bacteria in the gut microbiome of CD subjects. In the current study, matched oral and gut samples were taken and shown for the first time in CD subjects that *S. salivarius* were of similar strains. This increases the likelihood that the oral microbiome was the source for ectopic gut colonization in CD. This study also found that subjects in the active phase of disease were more likely to have ectopic gut colonization from the oral microbiome. A previous study found that oral microbes can colonize the gut after diarrheal episodes [15]. The authors hypothesized that the loss of gut microbes during diarrheal episodes reduces bacterial competition, this coupled with a transient increase in oxygen, allows for the ectopic colonization of oral microbes. Diarrhea is a major symptom during the active phase CD, suggesting that the oral microbiome can serve as a reservoir for pathogenic recolonization of the gut. A factor in ectopic gut colonization is the use of proton pump inhibitors which decreases the gastric acidity, allowing for ectopic gut colonization of oral microbes [22]. However, this study did not find that the use of proton pump inhibitors and low gastric acid state increases the likelihood of ectopic gut colonization. A limitation of the study is the small number of subjects in which concordant gut and oral strains were found. Furthermore, although not statistically significant, all CD subjects with concordant gut and oral *S. salivarius* were Han Chinese. Therefore, it cannot be ruled out that racial differences [11] contributed to the finding of ectopic gut colonization. Larger follow up studies collecting both oral and gut samples will be required to expand upon this finding.

The gut microbial species found at high abundances (> 5%) in both CD and healthy controls in this study were similar to previous studies employing WGS [10, 23]. Contrary to previous studies, this study did not find significant reduction of alpha-diversity in the gut microbiome of CD subjects [24, 25]. However, most of these studies used 16S rRNA sequencing, which may be the reason for the difference. Additionally, another aspect of the study was that most of the CD subjects were in remission (CDAI < 150) and well-controlled with medication, which may be a reason for their microbiome being less divergent from control. However, this would mean that any changes detected are more likely to be key changes driving microbiome dysbiosis.

The differentially abundant species found in gut microbiomes here were similar to previous studies. The depletion of butyrate-producing species such as *F. prausnitzii* and *R. inulinivorans* has been described in CD subjects [26]. *F. prausnitzii* in particular has been proposed to have protective effects in CD [27, 28], as the butyrate produced by these microbes aid in mucosal barrier function and maintenance of gut health [23]. Although *Prevotella sp.* has been studied extensively and some species have been shown to induce colitis in mice, it has yet been found to be associated with CD in humans [29]. In this study, it was found that *P. copri* was detected in a discreet cluster of healthy controls, similar to the previous WGS study [10]. This suggests that *P. copri* may be protective in some individuals against CD instead. *A. senegalensis* is a newly delineated species [30], which has yet to be implicated in CD. A related species, *Alistipes putredinis* has been described to contribute to transcriptional pathways in IBD [23]. Interestingly, it was also found to be significantly depleted in CD subjects in a previous WGS study [10] warranting further investigation. Additionally, a commonly implicated microbe *Ruminococcus gnavus* [23, 31] was found to be significantly enriched in CD subjects in this study. A recent mechanistic study into *R. gnavus* showed that it produces a complex polysaccharide that can result in the pattern of inflammation seen in CD [32]. Another species implicated in the pathogenesis of CD is *E. coli*, which was found to work in conjunction with other microbes as well as by exhibiting virulence features in CD subjects [31]. Although *Clostridium nexile* was found to be enriched in CD subjects in this study, it was found to be decreased in previous studies [33]. Comparing this to another WGS study, *C. nexile* was also found to be enriched in CD subjects, although not significantly [10]. *C. nexile* is able to produce short-chain fatty acids which decreases inflammation and alleviate colitis in experimental models [34]. However, the exact mechanism of that action is still unknown and does not explain why *C. nexile* was enriched instead in CD. This suggests that *C. nexile* has a geographically or ethnically specific role and its function warrants further investigation.

Experts in the field have suggested of use the salivary microbiome as a non-invasive tool in the diagnosis of CD [18, 19]. This study did not find any significant differences between the oral microbiome in CD and controls after adjustment, which could be due to the CD subjects being under treatment, with microbiome alterations due to medications rather than a signature of disease. However, when examining the genus differences, the study found similar enrichment of *Streptococcus,* and depletion of *Haemophilus* and *Prevotella* in CD subjects from previous 16S studies [18, 19]. Additionally, the ability to detect changes at species level provided better resolution than previous studies showing that the enrichment of *Streptococcus* genus involved several species such as *S. anginosus*, *S. oligofermentans*, *S. vestibularis*, *S. cristatus* and *S. salivarius*, while the depletion of *Prevotella* involved *P. salivae* and *P. intermedia*.

Another interesting finding from the salivary analysis is the enrichment of *Human Herpesvirus 4*, also known as Epstein-Barr virus (EBV), found in CD subjects. Recent studies have found a significant correlation between EBV presence in the gut with clinical disease severity [35]. This could be due to increased susceptibility of the CD patients on immunosuppressive treatment and the ability of EBV to induce inflammation. Being able to detect this in the oral microbiome may allow it to serve as a marker for disease severity.

The number of microbial pathways detected in this study was similar to a previous WGS study which detected 255 pathways in CD subjects [10]. The microbial pathway analysis found that 4 pathways in the gut microbiome with higher relative abundance levels found in CD compared to control are related to arginine biosynthesis. The arginine pathway is a major contributor to host inflammatory processes via inducible nitric oxide (NO) production, with NO providing protective cytostatic/cytotoxic antimicrobial action [36]. Administration of L-arginine has even been shown to reduce intestinal inflammation and pathology [37]. However, the specific role of microbial arginine metabolism has yet to be explored in the context of CD. It could be that these bacteria proliferate in the arginine poor environment in CD subjects as they are able to produce their own arginine. Additionally, isoprene biosynthesis was also found to be at higher relative abundance levels in CD subjects. The increase of isoprene in expired air from IBD subjects has been linked to the activity of disease, and is at a higher level than healthy individuals [38]. The microbial dysbiosis in CD favors a shift towards arginine and isoprene forming microbes providing a possible target to modify the microbiome.

## Conclusions

The use of metagenomics has highlighted the relationship of the gut and oral microbiome in well-controlled CD subjects. The study found the evidence of ectopic gut colonization by oral bacteria in CD subject during active disease, suggesting that the oral microbiome could be a reservoir for pathogens in CD patients. In addition to corroborating previously implicated gut microbial differences such as *R. gnavus* and *F. prausnitzii,* this study also detected the depletion of the newly described *A. senegalensis* not previously implicated in CD. Moreover, microbial arginine and isoprene pathways were found to be commonly present in CD gut microbiome, indicating that further study in this area is warranted.

## Methods

### Subject inclusion and recruitment

The study was conducted at the National University Hospital, Singapore from May 2017 to January 2019. Subjects with CD (n = 25) were recruited from the IBD clinic, Division of Gastroenterology & Hepatology. Healthy controls (n = 25) without any signs and symptoms of CD were age, sex and ethnicity matched and recruited from the Dental Centre, University Dental Cluster. The control subjects presented at the Dental Centre for routine dental care such as check-up, cleaning and minor restoration. Demographic information, history of tobacco use, other co-morbidities, current drug regimen and current disease activity were recorded. Current disease activity was calculated using the Crohn’s Disease activity index (CDAI) as well as the presence of common signs and symptoms (Diarrhoea/abdominal pain/blood-in-stool/fever and fatigue) [39]. Subjects who had antibiotic/probiotic/prebiotic use within a month, actively treated for a malignancy with chemotherapy, or diagnosed with an indeterminate colitis were excluded from the study. Non-CD controls were further required to have no known gastrointestinal signs and symptoms such as diarrhoea, abdominal pain and blood-in-stool.

The study received ethics approval from the institutional review boards of the National Healthcare Group, Singapore (DSRB reference E/2016/01,285). Informed consent was obtained from all participants. Additionally, all experiments were performed in accordance with relevant guidelines and regulations.

### Oral condition of subjects

Oral involvement of CD is well documented; with studies reporting between 20 and 50% of cases exhibiting oral manifestations including aphthous ulcers, linear deep ulcers, mucosal tags, mucosal “cobblestoning”, mucogingivitis and lip swelling. The presence of these conditions can affect the oral microbiome [40]. In order to detect differences in the oral microbiome that are key drivers of CD progression, subjects exhibiting oral manifestations were excluded for this study. Other conditions that can affect the oral microbiome such as the presence of caries and periodontal disease were also recorded at sample collection. This was verified by an oral examination conducted by a dentist under artificial light.

### Sample collection

Saliva was collected for the examination of the oral microbiome. Subjects were told to refrain from eating, smoking and dental procedures one hour prior to the collection. At least 5 ml of resting whole saliva was collected using the OMNIgene Discover Kit 505 (DNA Genotek, Ottawa, ON, Canada) according to manufacturer’s instructions.

Fecal sample was collected for the examination of the gut microbiome. Subjects were instructed and issued the OMNIgene•GUT (OMR-200) (DNA Genotek, Ottawa, ON, Canada) kit for sample collection. This was completed within two weeks of the saliva collection.

The oral and fecal samples were stored at room temperature as per manufacturer’s recommendation and sent for DNA extraction within 4 weeks from collection.

### DNA extraction and purification

Community DNA was extracted from saliva and fecal samples after mechanical lysis via bead-beating using Exgene™ Clinic SV Mini kits (GeneAll Biotechnology, Dongnam-ro, Seoul, Korea), and QIAmp® FAST DNA Stool Mini kits (Qiagen, Hilden, Germany) respectively according to manufacturer’s instructions. Extracted DNA was purified using AMPure XP beads (Beckman Coulter, Indianapolis, IN, USA). The quantity and quality of DNA was examined using a NanoDrop 8000 Spectrophotometer (Thermo Fisher Scientific, Carlsbad, CA, United States). Extracted DNA samples were stored at -80 °C for up to 6 weeks prior to library preparation.

### Library preparation and Illumina sequencing

Indexed sequencing libraries were prepared using QIAGEN® QIAseq FX DNA Library Kit (Qiagen, Hilden, Germany) following manufacturer’s instructions and sequenced as paired-end reads 2 × 151 bp on the Illumina HiSeq 4000 sequencer (Illumina, San Diego, CA, USA). On average, 15.5 million raw read pairs were obtained for each sample.

### Sequence data processing

Sequenced reads for each library were de-multiplexed into individual fastq file, and analysed using a pipeline (https://github.com/gis-rpd/pipelines/tree/master/metagenomics/shotgun-metagenomics) for processing paired-end shotgun metagenomic sequencing data. Firstly, raw reads was analyzed using Skewer to trim-off adapter sequences and low-quality bases from each read [41]. After trimming, reads were decontaminated to remove genomic sequences from the human host by using BWA-MEM to map reads against the hg19 reference [42]. The remaining reads were regarded as likely of microbial origin (average = 7 million) and were used as input for subsequent taxonomic and functional profiling.

### Taxonomic profiling

The MetaPhlAn2 software was used to profile the taxonomic composition of the microbial communities from the post quality-filtered reads [43]. Reads with sequences that matched microbial clades were used to normalize and calculate relative abundance for taxa from kingdom to species ranks. For reducing noise from false positive identifications, taxa with total relative abundance < 0.1% were excluded from further statistical analysis.

### Strain typing

To examine the relationship of the oral microbiome to the gut microbiome, strain analysis on *Streptoccous salivarius*, a predominantly oral microbe, was conducted on all gut and oral samples using StrainPhlAn. Using the reads mapped against the marker database of MetaPhlAn2, we extracted the clade specific markers of *S. salivarius*. Subsequently multiple sequence alignment of the marker sequences of *S. salivarius* strains was performed among the gut and saliva libraries of samples as well as the reference genome (NC_017594), before building the phylogenetic tree [44].

### Statistical analysis

#### Principal coordinates analysis (PCoA)

Bray–Curtis distance was calculated for PCoA analysis based on the species profiled in the sample set, while the multi-dimensional scaling (MDS) dimensions 1 and 2 were used to visualize the level of similarity between samples based on their microbiome composition.

#### Diversity analysis

The Vegan package in R was used to calculate Shannon and Simpson’s diversity values at the species level. Subsequently, ggplot2 was used to generate diversity plots. Wilcoxon test in R was used to test for significant difference in the medians of Shannon diversity values between case and control groups.

#### Association analysis for taxonomic abundances with Crohn’s disease

The Wilcoxon test in R was used to compare the median relative abundance in the cases with Crohn’s disease (CD) versus the non-diseased control group for a significant difference. To analyze the direction of the association, the “alternative” function was used in the model.

#### Multiple testing correction

We adopted Benjamin Hochberg’s false discovery rate method to correct for multiple testing at a significance threshold of 5%.

#### Pathway analysis

The HMP Unified Metabolic Analysis Network (HUMAnN2) program was used to determine relative abundance of microbial pathways in gut and saliva microbiomes of CD and control groups. The default Kyoto encyclopedia of genes and genomes (KEGG) orthology catalogue was used as the pathway reference. Subsequently, we extracted the total pathway abundance contributed by the genes present in every taxa of the community for analysis. For association, the Linear discriminant analysis Effect Size (LEfSe) software recognized the relative abundance of each pathway as the feature to test for significant difference in the medians between the two groups. We used the default LDA scores ≥ 2 as the threshold for significance, and to highlight the significance of the association, a more stringent threshold at ≥ 3 was also used for reporting [45].

## Supplementary Information

**Additional file 1: Table S1.** Pairwise distance between S. salivarius strains gene markers analysis.

## Data Availability

Whole-genome sequencing data that support the findings of this study are available in the European Nucleotide Archive with the primary accession code PRJEB39813.
